# Examining the effect of the individual characteristics of implementers and the interaction of multiple relationships on the structure of psychosocial intervention teams

**DOI:** 10.1186/s13012-020-01032-9

**Published:** 2020-08-28

**Authors:** Ignacio Ramos-Vidal, Jorge Palacio, Ilse Villamil, Alicia Uribe

**Affiliations:** 1 Department of Social Psychology, University of Seville, Seville, Spain; 2 Faculty of Psychology, Universidad del Norte, Barranquilla, Colombia; 3 Research Group CAVIDA, Universidad Pontificia Bolivariana, Montería, Colombia

**Keywords:** Implementation, Information exchange, Mental health, Network analysis, Psychosocial intervention, War victims

## Abstract

**Background:**

Teams’ structure may undergo modifications due to the individual attributes of actors and collective-level variables. This research aims to understand the effect of extensive experience working in the program and the simultaneous interaction among different relationships in the network structure of a team of implementers. The Psychosocial Care Program for Victims of Conflict is implemented by psychologists, social workers, and community advocates.

**Methods:**

A cross-sectional study was carried out. Multivariate analysis, quadratic assignment procedures, and graphic visualization are used to (a) determine how seniority affects the professionals’ level of centrality in the program and (b) clarify how the interaction among professionals favors new relationships.

**Results:**

Longer-lasting professionals in the program report stronger network bonding, predisposition to work, and information exchange. The nonparametric permutation test indicates an intense association between the information requests submitted and received and between the predisposition to work network and the network of received information requests. The results are discussed to optimize the teams implementing the intervention programs.

**Conclusions:**

Network analysis is a powerfull tool to evaluate program implementation processes. Analyzing the interactions among multiples relationships that emerge between members of multidisciplinary teams allows knowing how certain relationships (e.g., information exchange) triggering other kind of relationships (e.g., users referral). The implementers who have been collaborating in the program for a long time are key informants who can facilitate the process of adaptation of newly incorporated professionals.

Contributions to the literature

- Empirical evidence supports that implementation process needs to be analyzed from a structural perspective, by examining multiples kinds of interactions among the implementers.
- Seniority of implementers within the program is a key variable to understand the positions holding by the professionals in multiple networks.
- Longer-lasting professionals are identified as key informants by newcomers and may develop the role of promoters of program changes.
- Relationships that connect the professionals in the implementation process are interwitned, and the information exchange has the potential of triggering other relationships (e.g., users referral) wich are essentials for service delivery.

## Introduction

According to data of the Single Registry of Victims (SRV) launched by the Colombian government, since 1985, the armed conflict has left almost eight million victims of different criminal acts [1]. Throughout the country, eight million forced displacements, 984,408 homicides, 328,380 threatened people, 92,946 victims of terrorist acts, 34,404 abductions, and more than 10,000 anti-personnel mine victims have been reported. These numbers mean that at least 16.5% of the country’s population has suffered from the effects of the conflict.

In Córdoba, where the present study was conducted, there are 400,000 victims. This number reflects that more than 20% of the local population has been a victim of war. Empirical evidence suggested that people that have suffered episodes of violence (kidnapping, torture, massacres, or forced displacement) exhibit mental health problems with a prevalence ranging between 1.5 and 32.9% [2]. According to the results derived from a systematic review [3], the main mental health problems identified in populations affected by wartime violence are post-traumatic stress disorder (9%), major depressive disorder (5%), and generalized anxiety disorder (4%). Although the Colombian Government has developed several initiatives to improve the mental health of war victims, these efforts seem insufficient due to dimensions of the problem and the low level of adjustment of the interventions regarding the population needs. The magnitude of these numbers emphasizes the need to implement effective intervention programs to impact the psychosocial well-being of the population.

This research examines different types of relationships among professionals who implement a program that provides psychosocial care to victims of war in Colombia. In 2013, the Ministry of Health and Social Protection of Colombia launched a program known as the PAPSIVI (Comprehensive Program of Psychosocial Care and Health for Victims of Conflict) to meet the psychosocial care demands of the victims. In departments such as Córdoba, governance is decentralized, with the Health Development Secretariat being the entity in charge of implementing this program. The decision to explore the implementation process of this specific program was adopted due to (a) the nation-wide coverage of the program, (b) the multilevel nature of the intervention, and (c) the lack of empirical evidence regarding to the intervention effectiveness. To benefit from the PAPSIVI, users must be previously registered in the SRV. In 2017, the PAPSIVI attended to 8803 of the 9780 targeted victims for that year in Córdoba. This figure represents almost 90% coverage. The professionals implementing the program in the municipalities (in the Colombian context, a municipality is a geographical demarcation that may include small and medium-size cities as well as rural communities) are composed of psychologists (*n* = 26), social workers (*n* = 26), community advocates (*n* = 13), a liaison nurse, a physician, and six (*n* = 6) people who constitute the administrative staff, altogether providing psychosocial care to individuals, families, and the community. The interventions are provided by teams consisting of psychologists, social workers, and a community advocate who serve between one and three municipalities, depending on the number of victims. The rest of professionals (liaison nurse, physician, and administrative staff) develop other task related with the program but not centered in provide healthcare itself for this reason they are not included within intervention teams. The liaison nurse and the physician are dedicated to evaluate particular cases of users that present special needs (for example severe mental illness) that requires be referred to mental health services. The administrative staff receipts the documentation of the users and solves questions associated with the institutional requirements to participate in the program.

In healthcare settings, the intervention process is usually delivered by groups of professionals who work in a collaborative fashion to improve the service quality [4]. The literature shows the importance of work teams for providing high-quality care to users and patients [5]. There are several types of work teams depending on their structure, composition, functions, and task distribution [6]. Cross-functional, self-directed, and multidisciplinary are some of the many forms teams can adopt in organizational environments [7]. A cross-functional team is a group of professionals with different background and experiences which working toward a common objective; self-directed teams are conceptualized as a set of individuals who share the responsibility to develop specific tasks and work with low level of supervision; and multidisciplinary team is a group of people, with different academic backgrounds and professional experiences, who work together for achieving a common goal [8]. In the specific context of the program examined in this work, teams operate as multidisciplinary team care (MTC) in which professionals from a range of discipline (in this case psychologists, social workers, and community facilitators) work together to deliver comprehensive care that addresses as many of the patient’s needs as possible [9]. The users of the PAPSIVI exhibit psychosocial problems, difficulties of adjustment to the community settings they inhabit, and at collective level, those contexts presents several barriers to overcome vulnerability conditions. Considering these factors, multidisciplinary team care is considered an adequate design to solve the variety of demands affecting victims attended by this initiative.

Psychologists intervene at the individual level and to a lesser extent also perform family intervention. They focus on the initial diagnosis of the user and on designing an individual work plan. The intervention process consists of the following phases: (a) contacting, (b) diagnosis to design the individual action plan, (c) the development of the action plan following the PAPSIVI guidelines, and (d) a final closing session. Each intervention consists of six to eight sessions, and the number and intensity of sessions may vary, depending on each case (https://www.minsalud.gov.co/sites/rid/Lists/BibliotecaDigital/RIDE/DE/PS/Protocolo-de-atencion-integral-en-salud-papsivi.pdf). Social workers intervene at the community level. They perform collective work-based therapies with small groups and mutual aid groups. Such activities are designed so that (a) the community can confer sense to the victimizing act and reduce the sense of guilty that many of them experience, (b) promote a sense of identity with the community, and (c) increase community cohesion and strengthen social capital. Advocates (a) identify victims in communities, (b) channel them to the program, and (c) assume accompanying roles for the rest of the professionals. Community advocates reside in the municipalities where they render their services and are victims themselves. This aspect facilitates access to the population and increases the ecological validity of the intervention [10]. Once the implementers are assigned to a team, and the team is assigned to attend to a community, in the first stage the community advocate is the first actor to access the community, usually a few days before the arrival of the rest of the team members. The objective of this first stage is to identify the users and maintain contact with community leaders to explain the activities the rest of implementers will carry out in the next days. Within the multidisciplinary care team, one professional (psychologist or social worker) act as team coordinator. The main functions of the coordinator is to (a) organize the visits to the users, (b) evaluate which cases requires combined attention with more than one professional and, at the end of the visit to the community (b) receipt information about incidences during the intervention process. If within the team there is a professional newly incorporated into the program, it is assigned to the community promoter who acts as a mentor during the first days working in the communities.

In the Department of Córdoba, there are a number of factors that influence the coordination between the professionals implementing the program and that may affect the effectiveness indicators. First, the heterogenous composition of the teams is important to consider. Each team is composed of professionals with different technical backgrounds, which makes it necessary to pay special attention to coordination [11]. Second, the high turnover of personnel participating in the program makes the average time of participation in the teams less than a year, which may be affecting the results of the intervention [12]. Third, the geographic dispersion of teams that render attention to distant municipalities makes coordination among professionals extremely important for obtaining positive results [13].

Program success depends on the proper coordination among the implementers [14]. Recent studies highlight the importance to deeply understand the interaction patterns connecting the implementers. The relational dynamic and the networks structure that support program development determine various implementation outcomes such as acceptability, appropriateness, adoption, feasibility, and fidelity [15]. Effective coordination requires that the professionals (a) exchange relevant information regarding their services, (b) refer users to other professionals in terms of demand, and (c) participate in meetings and activities to ensure coordination [16]. The lack of coordination among service providers is related to (a) poor intervention results, (b) poor use of resources, (c) duplication of efforts, and (d) low satisfaction levels of users [17].

Collaboration, information sharing, and the referral of patients among professionals are key elements in identifying and systematizing good intervention practices [18]. Intervention programs that regulate user referral often yield better results [19]. Under this logic, user referral and information exchange optimize the implementation of programs, and from an organizational perspective, these practices benefit the institutions that coordinate service provision [20]. Previous studies have suggested that user referral reduces the cost of the service provided and improves the quality of diagnosis and treatment [21]. The adoption of evidence-based clinical practices by healthcare professionals is affected by the ties maintained by implementers. Palinkas and colleagues note that practitioners who actively exchange information and professional advice are more likely to adopt good intervention practices [22].

Individual and organizational factors influence information exchange and user referral. Individual factors describe the characteristics of professionals that encourage or inhibit interactions with other professionals. Organizational factors refer to group variables that affect the relations among professionals. Among the individual factors are the seniority of the professional in the program, the professional profile, and the empowerment level in the workplace. Some studies suggested that seniority of implementers in the program is a key factor to understand the role of professionals in the implementation process. More experienced professionals develop deep understanding of the implementation process, serves as key informants of other team members, and increase the adherence of users to the program activities [23]. Some of the organizational variables that affect the coordination and referral of users are organizational size, the level of decentralization in decision making, and geographic dispersion [14].

It is essential to analyze the structure of interactions among implementers because some relationships encourage new interactions [24]. Therefore, analysis of the network in which professionals are integrated may be considered an interesting phenomenon from the organizational perspective. Implementers request information from other colleagues with whom they have previously exchanged information or referred patients [25]. To discern how multiple relationships interact, it is necessary to know the background of the contacts that link the professionals who implement intervention programs. Doing so will make it possible to identify the relationships whose activation is a priority for applying structural changes with a positive impact on the program outcome. It is also necessary to identify the individual variables with potential to explain the role and position of the implementers in networks of information exchange and user referral. By examining the individual and relational factors that influence information exchange and user referral, it is possible to understand the governing logic of the program’s implementation to increase the effectiveness of the intervention.

### Objectives of the current study

The general objective is to determine how individual and relational factors influence the structure of different relationships among PAPSIVI implementers in the Department of Córdoba. The specific objectives are as follows:

Objective 1. To analyze the structural features of six types of networks in which PAPSIVI implementers participate: (a) the recognition network that examines the degree to which implementers are able to recognize other professionals by name; (b) the predisposition to work network which aims to know the level of affinity between the program implementers; (c) the network of submitted information requests; (d) the network of received information requests; (e) the network of submitted user referrals; and (f) the network of received user referrals.

Objective 2. The impact of seniority (how long each professional has worked in the program) in the position held in the six evaluated networks is examined at the individual level.

Objective 3. The dependence among the six networks is analyzed at the relational level, evaluating overlaps and the role of (a) recognition networks, (b) the predisposition to work, and (c) information exchange (received and submitted information requests) in the user referrals (submitted and received).

## Material and methods

### Participants

A total of 49 of the 65 (78.4%) professionals who implemented PAPSIVI in Córdoba were interviewed. The majority were women (94.1%, *n* = 48). The participants included 18 psychologists (35.3%), 21 social workers (43.1%), and 10 community advocates (19.6%). The participants stayed in the program an average of 12 months (range = 1–47; SD = 10.7). However, this figure varied notably between each profile, with community advocates lasting the longest, with an average of more than two years implementing the program, compared to 6 months for psychologists and eight months for social workers.

### Design and procedure

The study is cross-sectional and exploratory and the data presented here is part of a broader study designed to understand the variables associated to the implementation process that could affect the program effectiveness. The protocol of this study was revised to and approved by the Center for Research, Development and Innovation of the Universidad Pontificia Bolivariana. The project that supported this research was approved by the institutional review board of the University. All study participants provided written informed consent.

The PAPSIVI coordinators in Córdoba were informed of the purpose of the study, and the research team signed a confidentiality agreement. The participants signed an informed consent form. The interviews were conducted in coordination meetings held periodically in the capital of Córdoba between September and December 2016 and lasted approximately 1 h. The information gathering process occurred as follows: (a) a member of the research team traveled to the monthly coordination meetings that bring together all PAPSIVI implementers; (b) then, the researcher presents the characteristics of the questionnaire indicating how it should be answered by giving concrete examples; (c) during the completion of the relational data, the researcher offers guidelines on how to respond to the socio-centric instrument. All the implementers were invited to participate; however, those that were displaced in remote rural communities for providing service during the coordination meetings do not participate in the study. The STROBE checklist http://www.equator-network.org/reporting-guidelines/strobe/ for observational cross-sectional studies is included as additional file.

**Instruments**

The questionnaire includes socio-demographic variables and indicators related to the time that the professionals have been working in the program, the characteristics of the service provided, and the number of municipalities served. It also incorporates open- and closed-ended questions to learn the potential advantages and disadvantages associated with coordination activities, information exchange, and user referral [16]. Social network analysis (SNA) helps to understand how certain relationships favor other interactions, showing the interdependence among different social systems composed of the same professionals [26, 27]. Some proposals show the interdependence among multiple interactions that shape the structure of socio-health professional teams [28]. Reciprocity is important in referring users and sharing information, which means that cohesion measures such as homophily, transitivity, and reciprocity explain much of the structural variability of networks [29, 30]. This shows interdependence among the characteristics of the micro-local units that constitute the networks (e.g., dyads) and the global network structure [31, 32].

The design of the network instrument was based in previous studies which suggests that when asking about specific exchanges within healthcare settings (such information exchange or patients referral), it is interesting to differentiate among when the exchange consisting in sending or receipting that petition [16, 18, 33]. To analyze the six relationships among the professionals, each professional was asked about the relationships he maintains with the other program implementers indicating whether he maintains or does not maintain a relationship in each of the six networks evaluated. In the cases of users referral network and information exchange networks (both received and submitted), implementers were encouraged to nominate only professionals with whom they exchanged information or referred users within the previous month. This decision was made in order to identify the most accurate structure of real interactions. The resulting output analyzed is a dichotomous matrix for each network. A socio-centric network design was chosen because it captures direct and indirect interactions and is the most advisable when the network composition is defined by formal limits; in this case, by being part of the team of implementers of the program [34].

### Data processing

The socio-demographic variables and the implication indicators in the program were analyzed with the statistical package SPSS® (Versión 24.0. Armonk, NY: IBM Corp). The adjacency matrices used were processed with UCINET 6.636 [35]. The structure and composition indicators of the networks were calculated with UCINET and subsequently processed in SPSS® for multivariate analysis. The visual representation of the graphs was performed with the NETDRAW application included in UCINET.

### Data analysis

Multiple structural parameters were calculated to evaluate (a) the centrality parameters of each professional (Indegree and Outdegree), (b) the global network cohesion (density, indegree and outdegree centralization, and average degree), (c) the number of subgroups identified through cluster analysis using the optimization procedure proposed by Glover [36], (d) the dyadic properties (homophily according to category and seniority), and (e) triadic (transitivity) properties that affect the structural features of complete networks [29, 30, 32, 37].

To measure the effect of the seniority of the professionals in the program in the position that they hold on the six networks evaluated, first, the sample was segmented into two groups according to the seniority level. The division of groups was established using the 50th percentile as the cut-off point, which is equivalent to 8 months working on the program. The first group (group 0) is composed of 23 professionals who, on average, have been working less than 8 months in the program. The second group (group 1) is composed of 26 professionals who have been in the program for more than 8 months. The outdegree and indegree in the six networks evaluated and the density of each actor’s egocentric network were calculated to discover—through *t* test—the role of seniority in the position held by professionals in the six networks [38].

To examine the degree of overlap and the level of association between the different networks, several nonparametric permutation tests were performed through the Quadratic Assignment Procedure (QAP) at the dyadic level [27, 39]. To analyze the overlap among the six networks, a correlation analysis was performed. To identify how different types of relationships influence user referral, two multiple regression models were performed at the dyadic level. The double Dekker semi-partialling (DSP) technique was used because it is advisable when there are high levels of collinearity and self-correlation between variables [31]. In model 1, the dependent variable is the network of user referrals received, and the independent variables are the networks of user referrals (submitted), information requests (submitted), information requests (received), recognition of professionals, and predisposition to work. In model 2, the dependent variable is the network of user referrals submitted, and the independent variables are the networks of (a) user referrals (received), (b) information requests (submitted), (c) information requests (received), (d) recognition of professionals, and (e) predisposition to work.

## Results

### Description of the networks evaluated

The first research objective is focused on analyzing the structural cohesion of the six networks evaluated. The analyzed networks vary according to global cohesion, number of components, and the micro-structural features at the dyadic and triadic levels. Table 1 shows the parameters of the six networks evaluated.

**Table 1:** Cohesion parameters of the six networks evaluated

Type of relationship	Network cohesion measures							
	Density	Ind. Cent.	Out. Cent.	Mean degree	Trans.	Number of clusters and model adjustment	Homophily (professional category)	Homophily (based on seniority)
Recognition	51.17%	28.6%	49.8%	25.55	36.64%	7 (*R*^2^ = .197)	.240	− .141
Preference to work	7.3%	18.1%	24.4%	3.51	7.11%	10 (*R*^2^ = .132)	.163	− .132
Information request (submitted)	8.5%	23.2%	23.2%	4.08	7.04%	16 (*R*^2^ = .199)	− .091	− .154
Information request (received)	7.14%	11.84%	26.73%	12.41	6.09%	6 (*R*^2^ = .074)	− .056	− .116
User referrals (submitted)	1.74%	6.72%	19.48%	3.31	9.57%	7^c^ (*R*^2^ = .138)	.171	− .024
User referrals (received)	1.45%	7.03%	13.41%	2.81	5.47%	8 (*R*^2^ = .182)	.176	− .031

*Ind*. *Cent*. indegree centralization, *Out*. *Cent*. outdegree centralization, *Trans*. transitivity

Except for the recognition network with a high density (above 50%), the rest of the networks have low cohesion, ranging from 1.4 to 8.5%. Examining the values of centralization, it is observed that the networks are decentralized in both the input and output nominations. The input nominations are less centralized than output nominations in all relationships. In the network for information requests (referred), this indicator shows that a quarter of professionals make information requests to other colleagues and that, simultaneously, such requests are received by the same number of members. In the network of information requests received, the difference between input and output centralization implies that 26.73% of professionals request information from other colleagues and that those requests are received by 11.84% of the network components. This datum reflects that the request for information is typically focused on a few professionals. Similar differences between the centralization of input and output in the user referral networks (received and submitted) are observed. This finding suggests that between 15 and 20% of professionals refer users to other professionals, whereas the professionals who assume such referrals do not exceed 7%.

**Fig. 1:**
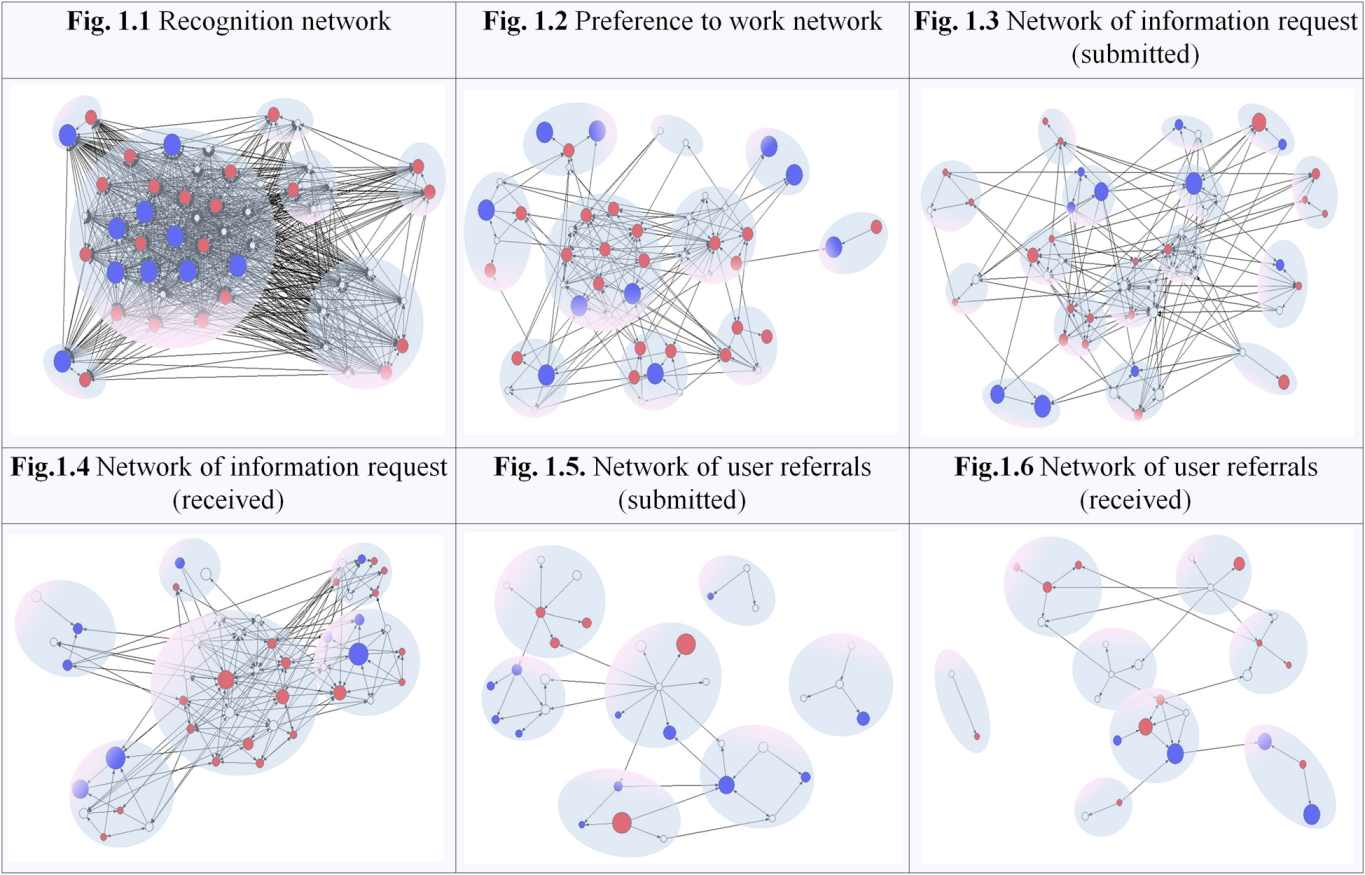
Graphs of the six networks. Node size represents time working in the program and node color denotes professional category (white = psychologists; red = social workers; blue = community advocates). Isolated nodes have been deleted from 1.4, 1.5, and 1.6

The mean nodal degree shows a similar trend across all networks. With the exception of the recognition network, which showed a high density, the rest have low transitivity that barely reaches 10%, which reflects low structural integration. This finding is shown in Fig. 1, in which the structure of the six analyzed networks is presented, identifying the clusters detected, and distinguishing the attributes of the actors by the color and size of the nodes.

There are six to 16 cohesive subsets. The information request network has the most groups, showing a high degree of fragmentation. Fifteen isolated nodes have been deleted from the user referrals network to calculate the number of clusters using the Tabu Search procedure [36].

Homophily values range between − 1 (pure homophily) and + 1 (pure heterophily). According to the professional profile, there is a moderate heterophilic tendency, except for the information request networks (received and submitted), in which a slight homophilic tendency is noted, with professionals of the same category exchanging information. It is possible to observe this tendency in Fig. 1(3 and 4), in which several of the clusters are formed of implementers of the same category. If this tendency is analyzed according to the seniority in the program, all of the networks exhibit a homophilic tendency, presenting moderate negative values. This finding indicates that seniority in the organization is a determining factor in all relationships explored.

### The role of seniority in individual positions

The second research objective is to learn how seniority in the program affects professionals in the position that they hold in the different networks. The longest-lasting professionals within the program (group 1) presented more connections in the recognition and information request networks. In the rest, no significant differences were observed. To determine the effect of seniority on individual positioning in the two groups, a *t* test was performed, discerning between the indegree value, the outdegree value, and the ego-centered density in the six networks. Table 2 shows the results of the T test analysis in the networks evaluated.

**Table 2:** *T* test to show the mean difference according to the time working in the program in the centrality parameters of the six networks

Type of relationship	T test indicators assuming equal variances						
	Group	Mean	Mean Difference	CI: 95% (Inf.)	CI: 95% (Sup.)	t	*p*
Outdegree (recognition)	0	38.04	− 24.69	− 36.42	− 12.96	− 4.306	**.0001**
	1	62.73					
Indegree (recognition)	0	41.48	− 18.21	− 25.13	− 11.27	− 5.35	**.0001**
	1	59.69					
Outdegree (preference to work)	0	7.69	.728	− 3.34	4.81	.359	.721
	1	6.97					
Indegree (preference to work)	0	5.25	− 3.88	− 7.21	− .55	− 2.345	**.023**
	1	9.13					
Outdegree (information request submitted)	0	9.32	1.557	− 2.22	5.22	.855	.397
	1	7.77					
Indegree (information request submitted)	0	6.52	− 3.73	− 7.45	− .012	− 2.018	**.049**
	1	10.25					
Outdegree (information request received)	0	5.34	− 3.38	− 7.82	1.04	− 1.153	.131
	1	8.73					
Indegree (information request received)	0	7.06	− .146	− 2.89	2.59	− .107	.915
	1	7.21					
Outdegree (user referrals submitted)	0	1.53	− .383	− 2.52	1.16	− .359	.721
	1	1.92					
Indegree (user referrals submitted)	0	1.35	− .724	− 1.82	.371	− 1.33	.190
	1	2.09					
Outdegree (user referrals received)	0	2.17	1.372	− .404	3.149	1.544	.127
	1	.80					
Indegree (user referrals received)	0	.72	− 1.358	− 2.53	− .182	− 2.323	**.025**
	1	2.08					
Ego-centered density (Recognition)	0	.60	.013	− .016	.043	.931	.357
	1	.59					
Ego-centered density (Preference to work)	0	.18	− .063	− .196	.070	− .958	.343
	1	.24					
Ego-centered density (Infor. request submitted)	0	.26	.142	.053	.231	3.228	**.002**
	1	.12					
Ego-centered density (Infor. request received)	0	.18	.068	.005	.130	2.188	**.034**
	1	.11					
Ego-centered density (User referrals submitted)	0	.02	− .020	− .090	.050	− .574	.569
	1	.04					
Ego-centered density (User referrals received)	0	.13	.039	− .055	.113	.832	.409
	1	.09					

Group 0 = < 8 months working in the program (*n* = 23); group 1 = > 8 months working in the program (*n* = 26)

To calculate the network density of each professional (ego-network density), the ego-centered model was applied [38]. In this model, each actor is treated as an ego and the ego network is trait as if the rest of the network did not exist so that ties beyond alters have no effect. Hence only are considered alter-alter ties as originally is suggested in Burt’s seminal work [38].

The most senior professionals in PAPSIVI are those who (a) know more colleagues, (b) have more nominations in the predisposition to work network, (c) receive more information requests from other colleagues, and (d) receive more referred users. However, seniority is not an important factor when determining the position that professionals hold either in the network of user referrals (submitted) or in the information requests network (submitted). In analyzing the differences in ego-centered density in the information exchange networks (both received and submitted), it is observed that the group with less time in the program presents comparatively denser networks than those of the longest-lasting group. In the rest of the networks, there are no significant differences between the two groups.

### Relational predictors of user referrals

The third objective seeks to identify whether relations of dependence among the six evaluated networks exist, specifically to determine how the analyzed multiple interactions influence user referrals (submitted and received). For that end, the association between the different networks is first examined through the QAP nonparametric permutation test [27]. Table 3 presents the results.

**Table 3:** QAP correlation coefficients

N°	Type of relationship	1		2		3		4		5		6	
		*r*	*p*	*r*	*p*	*r*	*p*	*r*	*p*	*r*	*p*	*r*	*p*
1	User referrals (submitted)												
2	User referrals (received)	.283	.001										
3	Information request (submitted)	.227	.001	.218	.001								
4	Information request (received)	.266	.001	.202	.001	**.484**	.001						
5	Recognition	.124	.001	.104	.001	.255	.001	.231	.001				
6	Preference to work	.062	.016	.171	.001	**.342**	.001	.315	.001	.239	.001		

In terms of the overlap magnitude, when the value of the Pearson *r* coefficient ranges from 0.1 to 0.3, a small correlation is reflected, values between 0.3 and 0.5 indicate a moderate correlation, and values higher than 0.5 reflect a high correlation [26]

The QAP shows a significant but low-intensity association [*r* = (.227–.283); *p* < .001] between the user referrals networks, both submitted and received, and the information request networks, both submitted and received. On the other hand, the user referral (received) network has similar associations, although with a slightly lower intensity range [*r* = (.207–.283); *p* < .001]. In addition, the Jaccard coefficient (*JC*) was calculated to determine the percentage of coincident actors in the network of user referrals submitted and received, identifying an association of 17.2% (*JC* = .172; *p* < .0001). This indicator shows a low degree of overlap if is compared with the concurrence level in the information networks submitted and received, which doubles (35.3%) its value (*JC* = .353; *p* < .0001). These results indicate a low level of integration and correspondence between professionals who refer users and those who receive referrals.

Finally, a nonparametric permutation test was proposed at the dyadic level using two multiple regression QAP (MRQAP) models to determine how the predisposition to work and information exchange (information requests submitted and received) networks influence the user referrals (submitted and received). The DSP method described above was performed [31]. Table 4 shows the results of the two MRQAP models.

**Table 4:** MRQAP coefficients applying the double semi-partialling procedure

N°	Independent networks	Dependent network							
		Model 1 User referrals (received)				Model 2 User referrals (submitted)			
		B	β	p	S.E	B	β	p	S.E
1	User referrals (submitted)	.217	.238	.0005	.0237	–	–	–	–
2	User referrals (received)	–	–	–	–	.255	.232	.0005	.0237
3	Information request (submitted)	.042	.099	.0015	.0105	.048	.103	.0005	.0108
4	Information request (received)	.025	.055	.0175	.0108	.093	.183	.0005	.0118
5	Recognition	.002	.012	.3128	.0056	.013	.050	.0135	.0061
6	Preference to work	.046	.102	.0005	.0103	− .041	− .082	.0005	.0108
Model Adjustment		*R*^2^ = .119; Δ*R*^2^ = .117; Prob. = .00001				*R*^2^ = .139; ΔR^2^ = .137; Prob. = .00001			

S.*E* standard error

In the first MRQAP model, in which the dependent variable is the network of user referrals received, the results show that altogether, networks that act independently are responsible for 11.7% of the dependent variance (*R*^2^ = .119; *ΔR*^2^ = .117; *p* < .00001), with the network of user referrals submitted being the variable with the highest predictive power (*β* = .238; *p* < .0005) and the other networks moderately contributing to the explained variance. However, the information requests network and the predisposition to work network also explain some variance of the dependent variable.

In the second model, the network of user referrals submitted acts as a dependent variable, whereas the network of user referrals received, the information exchange networks (received and submitted), the recognition network, and the predisposition to work network act as independent variables. The model summary indicates that the independent variables altogether account for 13% of the dependent variance (*R*^2^ = .139; *ΔR*^2^ = .137; *p* < .00001). As with the first model, the network that accounts for the largest proportion of variance is the network of user referrals received (*β* = .232; *p* < .0005). It is remarkable that the information exchange networks, particularly the network of information requests received, significantly contribute to explaining the variance of the dependent variable (*β* = .183; *p* < .0005). This finding demonstrates the connection between information exchange and user referral.

## Discussion

This research examines different types of relationships among professionals who implement a program that provides psychosocial care to victims of war in Colombia. The literature notes that the relationships among implementers have a direct effect on service quality and user satisfaction [17, 19]. To analyze these interactions, several SNA techniques are used (a) to perform descriptive analyses of the relational context of the professionals implementing the program (objective 1), (b) to determine the effect of certain characteristics such as seniority in the program when referring users (objective 2), and (c) to analyze the associations among different relationships (objective 3).

The cohesion measures examined yield interesting results. With the exception of the recognition network, which shows high cohesion (51.2%), as expected due to the number of implementers of the program, the rest of the networks show low cohesion. This indicator highlights the need to develop strategies to increase interactions among professionals in certain relationships. On the other hand, the moderate values in the centralization parameters show an equal distribution in the ties among the professionals; high values in the centralization parameters would show that a small proportion of actors are those with a greater proportion of relationships, possibly reflecting an unbalanced structure in establishing contacts. Work teams in which the network structure is decentralized show better performance compared to highly centralized teams [40]. In the particular case of this program, coordination and administrative staff may activate communication channels in order to increase interactions between MTC’s to promote the discover and dissemination of good intervention practices and lessons learned. The data reveal that to strengthen the structure of the teams, each network should undergo differential modifications. It is advisable to increase cohesion in the recognition network, which would be positive if most professionals knew each other. The same occurs in the information exchange and user referral (issued and received) networks, in which the cohesion levels show that there is low activity in both interactions. These moderate levels of exchange are partially explained by the configuration of the teams, but it is necessary to increase the interactions in both relationships, which are essential to providing quality service [18, 20]. Regarding the number of groups, it is possible to identify multiple groups (between six and 16) in all networks, even in the recognition group, which is the densest. This analysis makes it possible to identify subgroups of professionals who preferentially relate to each other [36]. The large number of identified groups shows several subgroups that articulate the structure of the network. This indicator is possibly a consequence of the geographical dispersion between the municipalities where the teams intervene and the high personnel turnover that determines the stability and the global cohesion in the networks.

These results may contribute to improving the effectiveness of the PAPSIVI and the quality of service. The analysis of social structures such as those that constitute teams that implement intervention programs is the most appropriate path to understanding the dynamics of cooperation within the teams [41]. Certain individual variables determine the place that a professional holds within a work team. In this research, the time that the professional has been working in the program is stressed as the most determining factor in explaining the relational context of the community interventors. This result may partly be explained by the accumulated experience, which makes these professionals, particularly the community advocates, who have a link between the program and the community, play a prominent role, ensuring that the professionals who have just joined in the program may receive proper instructions. As commented previously, community advocates are a key piece in the implementation process. They (a) present the program goals to community authorities, (b) reduce the initial uncertainty associated to the program activities and, in parallel, and (c) increase the rate of participation and adherence to the program. On the other hand, the high discontinuity in recruitment is worrisome; 46.9% of the study participants have been working in the program for less than 8 months. This aspect makes (a) strengthening teams, (b) proximity and access to the community, and (c) the extraction and dissemination of good practices difficult. There are several factors that may explain the overall low rate of seniority of PAPSIVI implementers. Psychosocial interveners are in continuous contact during the attendance process with people and communities that have suffered severe episodes of trauma and victimization. This fact produces that some professionals empathize with the users and, in some cases, develop high levels of fatigue, stress, and anxiety that motivate their decision to leave their work. In this line, previous studies suggest that healthcare professionals could be considered “second victims” as a consequence of the effects derived from the prolonged exposition to the users’ experience of trauma [42]. Another factor contributing to the staff turnover is that the hiring process in some periods is discontinued due to lack of financial resources and budget constraints. This causes, given the uncertainty about their future, some professionals to leave the program looking for more stable contracts in other organizations. To reduce implementers’ turnover, program managers may develop specific actions to increase motivation and satisfaction. Increasing motivation and satisfaction has proved effective in reducing turnover in healthcare professionals [43]. Achieving the stability of program implementers is a key factor having into account that experimented professionals may exert influence on the behavior of users with regards to the program, ramping up the adherence of users to the treatment [44].

The last purpose is to examine the interdependence among the different evaluated networks, assuming that when analyzing different relationships among the same set of actors, there is always a certain degree of overlap. Therefore, by analyzing the multiplicity, the extent to which one or more types of interaction may lead to new interactions among professionals can be determined [27]. In general, a range of moderate correlations is noted (*r* < .3), particularly among dyadic relationships. However, an interesting piece of information was found when observing the correlations between the information exchange network (submitted) and the rest of the networks.

The level of correlation between information requests submitted and received is high, which is to be expected, given the latent effect of reciprocity [29, 30]. However, there is a moderately high association between the predisposition to work network and, to a lesser extent, with the user referral networks. These results and, to a lesser degree, those expressed by the MRQAP models seem to indicate that associations between the analyzed networks exist and that information exchange plays a key role in both the selection of other professionals for work and the decision to refer users to other colleagues. The results shown suggest that, despite the multidisciplinary nature of the teams that implement the program, it is necessary to promote internal communication and develop protocols for the teams to work collaboratively to take advantage of the synergy of each professional profile and offer comprehensive care at the individual, family, and community levels of intervention. This implies that the managers of the PAPSIVI must facilitate that the work carried out by the teams allow them to attend in an integral way to the multiple demands that the users of the program exhibit.

### Limitations

This research is cross-sectional; for this reason, we do not have information on the characteristics of the professionals who left the program before carrying out this research. However, some plausible explanations supported on the literature on the factors that explain high turnover in the PAPSIVI program are offered. To do this, first is needed to explain in brief why community advocates are the professional group with more seniority in the program. Community advocates have the condition of victims recognized by the Government and are registered in the SRV. In most cases, the criterion to select them is that they should reside in the same community in which the program is implemented. For this reason, they do not need to displace to other communities to work, which is easy for them to follow collaborating in the program. Another factor that could explain the long-time they have been working in the program is that they do not develop interventions; they connect the other members of the multidisciplinary team care (psychologists and social workers) with the potential users of the program in the communities. Due to their role as connectors, rather than as interveners, they do not suffer the harmful effects derived from providing care to victims who have suffered severe episodes of trauma. Finally, community facilitators are considered a vulnerable population due to their victim status. This makes that probably working in the program is not only a great opportunity, but the only one to earn an income and support their families. In contrast, psychologists and social workers maintain continuous and direct contact with victims. This may provoke high levels of fatigue and stress that may contribute to activate turnover intentions. Psychologists and social workers, in contrast to community advocates, have to travel to other communities to provide care. Considering the geographical dispersion that characterizes rural communities in the Department of Córdoba, this is an extra factor that may contribute to turnover intentions. Finally, psychologists and social workers are qualified professionals that have more opportunities to find job in the labor market compared with community facilitators.

## Conclusions

Structural analysis tools (i.e., SNA) are a great ally in designing, implementing, and evaluating intervention programs [22]. The centrality indicators provide information on the individual positioning of actors. Consequently, the analysis of these measures is valuable in identifying key actors who can (a) disseminate good intervention practices, (b) integrate isolated professionals holding peripheral positions, and (c) adopt early changes in the work logic to facilitate the acceptance of the rest of the members [33, 41].

The cohesion parameters provide a global picture of the different networks evaluated. These indicators offer important clues to understand the work dynamics of the teams and the effects that such dynamics produce on the effectiveness of the interventions. It is advisable to evaluate different types of relationships to adequately define the relational context of the program implementers. This analysis initially makes recommendations that advise the interactions between the team members to be increased in some cases, whereas in others, it is advisable to increase the contacts within the same groupings. The results of this research suggest, in agreement with previous studies, that the relationships established by the implementers and the relational structure that underlies such interactions affect the results of the implementation process [15]. For this reason, since the stage of intervention design, it is necessary to incorporate instruments that allow evaluating the different types of interactions that occur among implementers during the intervention process. The results of such structural evaluation will serve to adapt the design and composition of multidisciplinary care teams to meet the demands of users.

For program designers and evaluators, it is important to know what individual variables are associated with holding central positions in different networks. The data of this research show that the seniority of professionals in the program is a determining variable in explaining the number of contacts established in different relationships. It is advisable to consider the most experienced workers as potential allies in the implementation of programs. Finally, it is essential to reduce the high rates of turnover in the program among psychologists and social workers in order to guarantee the appropriation of knowledge and take advantage of the accumulated experience derived from the service provision [45]. The managers of the program should promote the continuous training of professionals, the improvement of working conditions, and the actions aimed at increasing empowerment in the workplace to reduce turnover rates [46].

The need to evaluate multiple relationships simultaneously is emphasized. This analysis may be structured in two stages. In the first, the objective is to determine the degree of overlap and interconnectedness among the different networks. This phase can be developed using dyadic QAP correlations, in which the level of association among networks would initially be known, and second, the Jaccard Coefficient (JC) would be examined to identify the dyadic proportion present in several relations simultaneously. In the second phase, the information gathered from the previous stage would be used to design MRQAP models to determine the extent to which different networks (individually or in aggregate) have the capacity to favor or explain the emergence of other kinds of instrumental relationships.

The implementation of an intervention program can be evaluated from a structural perspective [33]. To strengthen the structure of teams, it is essential to know both the characteristics and the roles played by professionals who hold influential positions in the networks [47]. The identification of these professionals can be a determining factor for introducing changes in the networks. In parallel, it is necessary to precisely define what types of relationships will be evaluated [48]. The choice of the type of relationship must be guided with regard to relevant relationships to explain the factors that determine the effectiveness of the interventions. Finally, it is necessary to evaluate multiple relationships simultaneously. In this manner, it is possible to examine how relationships, independently (or together with others), can favor new interactions. This makes it possible to have a more accurate view of the relational context of the teams that implement intervention programs, directing the design of strategies to optimize the implementation.

## Supplementary information

**Additional file 1.** STROBE Statement—Checklist of items that should be included in reports of cross-sectional studies. Note: An Explanation and Elaboration article discusses each checklist item and gives methodological background and published examples of transparent reporting. The STROBE checklist is best used in conjunction with this article (freely available on the Web sites of PLoS Medicine at http://www.plosmedicine.org/, Annals of Internal Medicine at http://www.annals.org/, and Epidemiology at http://www.epidem.com/). Information on the STROBE Initiative is available at www.strobe-statement.org.

## Data Availability

The dataset supporting the conclusions of this article is available in the ICPSR repository, [10.3886/E115230V1].

## Abbreviations

DSP: Double Dekker semi-partialling
JC: Jaccard coefficient
MRQAP: Multiple regression quadratic assignment procedure
PAPSIVI: Psychosocial Care Program for Victims of Conflict (acronym in Spanish)
QAP: Quadratic assignment procedure
SNA: Social network analysis
SRV: Single registry of victims
